# Clinical characteristics and risk factors of lower extremity amputation in the diabetic inpatients with foot ulcers

**DOI:** 10.3389/fendo.2023.1144806

**Published:** 2023-03-31

**Authors:** Hongping Gong, Yan Ren, Zhenyi Li, Panpan Zha, Raju Bista, Yan Li, Dawei Chen, Yun Gao, Lihong Chen, Xingwu Ran, Chun Wang

**Affiliations:** ^1^ Department of Endocrinology and Metabolism, Diabetic Foot Care Center, West China Hospital, Sichuan University, Chengdu, Sichuan, China; ^2^ International Medical Center Ward, Department of General Practice, West China Hospital, Sichuan University, Chengdu, Sichuan, China; ^3^ Department of Clinical Research Management, West China Hospital, Sichuan University, Chengdu, Sichuan, China

**Keywords:** diabetic foot ulcer, lower extremity amputation, foot gangrene, minor amputation, prior amputation, risk factor

## Abstract

**Objectives:**

To analyze clinical characteristics of the diabetic inpatients with foot ulcers and explore the risk factors of lower extremity amputation (LEA) in West China Hospital of Sichuan University.

**Methods:**

A retrospective analysis was performed based on the clinical data of the patients with diabetic foot ulcer (DFU) hospitalized in West China Hospital of Sichuan University from January 1, 2012 to December 31, 2020. The DFU patients were divided into three groups: non-amputation, minor amputation, and major amputation groups. The ordinal logistic regression analysis was used to identify the risk factors for LEA.

**Results:**

992 diabetic patients (622 males and 370 females) with DFU were hospitalized in the Diabetic Foot Care Center of Sichuan University. Among them, 72 (7.3%) (55 minor amputations and 17 major amputations) cases experienced amputation, and 21(2.1%) refused amputation. Excluding the patients who refused amputation, the mean age and duration of diabetes of and HbA1c the 971 patients with DFU, were 65.1 ± 12.3 years old, 11.1 ± 7.6 years, and 8.6 ± 2.3% respectively. The patients in the major amputation group were older and had longer course of diabetes for a longer period of time than those in the non-amputation and minor amputation groups. Compared with the non-amputation patients (55.1%), more patients with amputation (minor amputation (63.5%) and major amputation (88.2%)) suffered from peripheral arterial disease (*P*=0.019). The amputated patients had statistically lower hemoglobin, serum albumin and ankle brachial index (ABI), but higher white blood cell, platelet counts, fibrinogen and C-reactive protein levels. The patients with amputation had a higher incidence of osteomyelitis (*P* = 0.006), foot gangrene (*P* < 0.001), and a history of prior amputations (*P* < 0.001) than those without amputation. Furthermore, a history of prior amputation (odds ratio 10.194; 95% *CI*, 2.646-39.279; *P*=0.001), foot gangrene (odds ratio 6.466; 95% *CI*, 1.576-26.539; *P*=0.010) and ABI (odds ratio 0.791; 95% *CI*, 0.639-0.980; *P* = 0.032) were significantly associated with LEAs.

**Conclusions:**

The DFU inpatients with amputation were older with long duration of diabetes, poorly glycemic control, malnutrition, PAD, severe foot ulcers with infection. A history of prior amputation, foot gangrene and a low ABI level were the independent predictors of LEA. Multidisciplinary intervention for DFU is essential to avoid amputation of the diabetic patients with foot ulcer.

## 1 Introduction

Diabetic foot ulcer (DFU), a severe and devastating complication of diabetes mellitus, typically presented as ulcers, infection, or destruction of tissues of the foot (1). The global diabetic foot ulcer prevalence of DFU was about 6.3% (2). DFU has always been the leading cause of non-traumatic lower extremity amputation (LEA) in the world. The rate of LEA in the diabetes was were more than five times higher than those without diabetes (3). The LEA rates were quite different in the different countries. A study in China indicated that the overall LEA rate among the DFU patients was about 19.03%, with major and minor amputation rates of 2.14% and 16.88%, respectively (4). Between 2001 and 2010, the LEA rate of the hospitalized patients with DFU in the United States was approximately 16.5% (34.8% for major and 61.2% for minor amputations) (5). In Africa, about 15% of the DFU patients underwent major amputation (6). In France, a prospective study of 347 patients with the new-onset DFU from 2001 to 2003 showed that the rates of major and minor amputation at one year were 10% and 19%, respectively (7). Furthermore, disability after LEA had a negative impact on the quality of life of the DFU patients.

An investigation revealed that the patients who had experienced diabetic foot–related complications were 79% more likely to rank LEA as their greatest fear when compared with death (8). Therefore, correctly identifying risk factors and strengthening risk prevention and control, were very important for the diabetic patients. Peripheral arterial disease (PAD), osteomyelitis, gangrene, increased inflammatory biomarkers, and low hemoglobin (Hb) levels were considered as the risk factors of LEA (9, 10). However, the risk factors for LEA of the diabetic patients in different studies were not completely consistent. Therefore, we collected clinical information of the diabetic patients with foot ulcers admitted in the Diabetic Foot Care Center of West China Hospital during Jan 1,2012 and Dec 31,2020 to analyze the clinical characteristics of the DFU inpatients with LEA (major and minor amputations) and explore the potential risk factors of LEA.

## 2 Patients and methods

### 2.1 Research objects

This is a retrospective study. The clinical data of all consecutive patients who were admitted to the Diabetic Foot Care Center in West China Hospital of Sichuan University between Jan 1, 2012 and Dec 31, 2020, were collected. The study has been approved by the Institutional Review Board Committee of West China Hospital of Sichuan University Hospital (No.2012-119). The diabetic patients who had foot ulcers met the diagnostic criteria for diabetic foot (Wagner grade 1 to 5) according to International Working Group on Diabetic Foot (IWGDF) guidelines were included in the study (11). The diabetic patients with lower limb ulcers above the ankle joint, hand ulcers, gouty ulcers and cancerous ulcers were excluded. In addition, the foot ulcers were caused by long-term use of glucocorticoids and other non-diabetic related were also excluded. Major and minor amputations referred to amputation above and below the ankle, respectively (12).

### 2.2 Data collection and processing

Electronic medical records of all patients were reviewed. All data were collected from hospital information system. The clinical information of the patients with DFU consisted of age, sex, course of diabetes, body mass index (BMI), diabetic medication history, smoking and drinking history, previous foot ulcer and amputation history, diabetic chronic complications, comorbidities and physical examinations. The severity of the foot ulcers was classified on the basis of the Wagner grading system.

Baseline laboratory data – including fasting blood glucose (FBG), glycosylated hemoglobin (HbA1c), blood routine (Hb, platelet (PLT), white blood cell (WBC) count, neutrophil granulocyte percentage (NEUT)), coagulation routine, liver function, serum lipid profiles, serum uric acid (UA), serum creatinine, estimated glomerular filtration rate (eGFR) and serum C-reactive protein (CRP) were collected. The ankle brachial index (ABI), the ratio of the systolic pressure measured at the ankle to that measured at the upper arm, were recorded.

### 2.3 Definitions of diabetic chronic complications and comorbidities

Diabetic retinopathy was diagnosed by the optometrist or through ophthalmological reports. The diagnosis of PAD was confirmed based on ABI ≤ 0.9 and/or results of doppler ultrasound of lower extremities. Diabetic peripheral neuropathy (DPN) was diagnosed based on neuropathic symptoms (such as numbness, tingling, or burning feeling, muscle weakness, etc.) and physical examination (pinprick, temperature sensation, vibration perception, proprioception, 10-g monofilament, and ankle reflexes) (13, 14). Cardiovascular autonomic neuropathy (CAN) was determined by resting tachycardia (>100 bpm), orthostatic hypotension (a fall in systolic blood pressure >20 mmHg and/or diastolic pressure >10mmHg within 3 minutes of standing) in the absence of an appropriate heart rate response (15). Diagnosis of gastrointestinal autonomic neuropathy should be reserved for patients with gastrointestinal symptoms (e.g. gastroparesis, constipation, diarrhea) and normal gastrointestinal examination (16). The clinical diagnosis of bladder autonomic neuropathy was based on the presence of lower urinary tract symptoms (e.g. dysuria, frequency, urgency, nocturia, recurrent cystitis, as well as stress and urgency urinary incontinence) with a bladder color doppler ultrasound for residual urine, and urological conditions such as benign prostatic hypertrophy in men or gynecological disorders in women must be ruled out by appropriate testing (14). Chronic kidney disease (CKD) was classified into five stages based on the eGFR (G1: eGFR ≥ 90 mL/min per 1.73 m^2^, G2: 60 to 89 mL/min per 1.73 m^2^, G3: 30 to 59 mL/min, G4: 15 to 29 mL/min per 1.73 m^2^, G5: < 15 mL/min per 1.73 m^2^) (17). Coronary heart disease was defined as myocardial infarction, angina, percutaneous coronary intervention or bypass surgery. Diagnosis of osteomyelitis was usually based on imaging(foot X-ray and foot MRI) and probe-to-bone test, and bone biopsy or microbial cultures can be used if necessary (18).

### 2.4 Statistical analysis

Statistical analysis was performed using IBM SPSS 26.0 software for Windows (IBM Corp., 2019). Continuous variables were reported as mean ± standard deviation or median (interquartile range). Differences among three groups were assessed using one-way ANOVA with Bonferroni post-test or Kruskal–Wallis test when inhomogeneity of variance existed. Categorical variables were expressed as frequencies with percentage (%) and compared with the chi-squared test or Fisher’s exact test. Multivariate stepwise ordinal logistic regression was used to identify potential predictors for LEA. Validity of the ordinal logistic regression model was assessed with the test of parallel lines, and significance was confirmed by −2 log likelihood. For each of the candidate predictors, the odds ratio (*OR*) for the likelihood of amputation was calculated. The ABI was adjusted by multiplying by 10 so as to fit the clinical convention when the odds ratio was calculated and interpreted. For all tests, statistical significance was set at *P* < 0.05.

## 3 Results

### 3.1 Baseline characteristics

992 diabetic patients (622 males and 370 females) with DFU were admitted in the Diabetic Foot Care Center of West China Hospital during 2012 and 2020. Among them, 72 cases were amputated and 21 refused amputations. Excluding the DFU patients who refused amputation, 971 patients with DFU were analyzed in the study. Of the 72 patients with LEA, 55 cases (76.4%) received minor amputation and 17(23.6%) experienced major amputation, respectively. The mean age of the DFU patients was 65.1 ± 12.3 years old and the mean course of diabetes was 11.1 ± 7.6 years. The patients with major amputation were older and had a longer duration of diabetes than those with non-amputation and minor amputation. Only two of the patients with major amputations were female, and nearly two-thirds of the amputees were men. Approximately half of the non-amputated and minor-amputated patients smoked previously or currently, while in the major groups, the percentage rose to 76.5% (**Table 1**).

**Table 1:** Baseline demographic and laboratory data among the non-amputation, minor amputation and major amputation groups.

Factor	Non-amputation (N=899)	Minor Amputation (N=55)	Major Amputation (N=17)	*P* value
Demographics				
Age, yr	65.1 ± 12.3	62.9 ± 12.4	69.4 ± 9.9	0.146
Sex				0.086
Male	563	33	15	
Female	336	22	2	
BMI, kg/m^2^	23.3 ± 3.4^†^(*n*=780)	23.2 ± 3.3^†^(*n*=45)	23.3 ± 3.4	0.118
Smoking (current or ever)	462	27	13	0.113
Drinking (current or ever)	346	18	10	0.155
Hospital stays (day)	30(1-244)	57(8-251)	47(18-114)	<0.001
Diabetes-related characteristics				
Duration of diabetes, yr	11.1 ± 7.6	10.9 ± 7.3	8.5 ± 4.7	0.416^*^
ABI	0.97 ± 0.28^†^(*n*=539)	0.85 ± 0.33^†^(*n*=23)	0.76 ± 0.31^†^(*n*=9)	0.012
Ulcer area, cm^2^	4.0(1.3-12.0)^†^(*n*=710)	10.0(3.0-32.4)^†^(*n*=45) ^a^	10.3(3.3-30.0)^†^(*n*=13)	0.005^*^
Laboratory results				
FBG, mmol/L	9.0 ± 4.1^†^(*n*=680)	9.2 ± 3.8^†^(*n*=37)	9.9 ± 4.1^†^(*n*=13)	0.666
HbA1c, %	8.6 ± 2.3^†^(*n*=803)	8.8 ± 2.4^†^(*n*=50)	8.7 ± 1.9^†^(*n*=16)	0.897
Hb, g/L	114 ± 22^†^(*n*=854)	104 ± 25 ^a^	109 ± 23	0.004
PLT, ×10^9^/L	232 ± 108^†^(*n*=851)	266 ± 104	304 ± 143 ^a^	0.001
FIB, g/L	4.4 ± 1.5^†^(*n*=827)	4.9 ± 1.7 (*n*=54)	5.0 ± 1.8 (*n*=16)	0.019
WBC count, ×10^9^/L	7.8 ± 3.7^†^(*n*=853)	9.6 ± 5.1^†^ (*n*=54)	11.6 ± 6.1	0.001^*^
NEUT, %	68.5 ± 12.5^†^(*n*=806)	71.4 ± 16.8	81.0 ± 9.3^†^(*n*=15) ^a^	<0.001^*^
Albumin, g/L	36.4 ± 6.1^†^(*n*=892)	33.1 ± 7.3^a^	32.8 ± 6.1^a^	<0.001
TG, mmol/L	2.1 ± 1.5^†^(*n*=887)	2.2 ± 1.6	1.6 ± 1.0	0.315
TC, mmol/L	3.5 ± 1.4^†^(*n*=888)	3.0 ± 1.4 ^a^	3.0 ± 1.2	0.016
LDL-C, mmol/L	2.2 ± 1.0^†^(*n*=888)	1.9 ± 0.9^†^(*n*=54)	1.9 ± 1.0	0.148
HDL-C, mmol/L	1.09(0.87-1.42)^†^(*n*=888)	1.03(0.67-1.48)	0.93(0.69-1.29)	0.308^*^
UA, μmol/L	322 ± 110^†^(*n*=888)	276 ± 123^†^(*n*=54) ^a^	255 ± 133 ^a^	0.001
Creatinine, μmol/L	81.0(63.5-108.0)^†^(*n*=892)	68.5(55.5-91.8)^†^(*n*=54)	70.6(53.5-103.5)	0.152
eGFR, mL/mL.1.73m^2^	76.4 ± 31.4^†^(*n*=864)	86.3 ± 32.7^†^(*n*=53)	85.5 ± 36.2^†^	0.047
CRP, mg/L	10.0 (3.3-30.2)^†^(*n*=547)	24.3(4.9-105.9)^†^(*n*=28)	74.6(9.7-146.5)^†^(*n*=13) ^a^	0.002^*^

Values are presented as number, median (IQR), or mean ± standard deviation. BMI, body mass index. ABI, ankle-brachial index, FBG, fasting blood glucose. HbA1c, glycosylated hemoglobin. Hb, hemoglobin. PLT, platelet. PT, prothrombin time. FIB, fibrinogen. WBC, white blood cell, NEUT, neutrophil granulocyte percentage. TG, triglyceride. TC, total cholesterol. LDL-C, low-density lipoprotein cholesterol. HDL-C, high-density lipoprotein cholesterol. UA, uric acid. eGFR, estimated glomerular filtration rate. CRP, C-reactive protein. †Some cases are lacking data and the number of patients was shown in brackets. *, Kruskal–Wallis test. a, statistical significance compared with non-amputation group with Bonferroni post-test.

PAD was more frequent in the patients with minor (63.5%) and major (88.2%) amputations than those without amputation (55.3%) (*P* = 0.014). More than 95% of the DFU patients suffered from DPN. There was no statistically difference in the incidence of coronary heart disease, hypertension, diabetic retinopathy, DPN, CKD, hyperlipidemia and hyperuricemia among the three groups (**Table 2**).

**Table 2:** Comparison of diabetic complications and comorbidities among the non-amputation, minor amputation and major amputation groups.

Factor	Non-amputation (N=899)	Minor Amputation (N=55)	Major Amputation (N=17)	*P* value
Retinopathy				0.970
Yes	351	20	6	
No	474	28	9	
Diabetic peripheral neuropathy				0.372
Yes	848	54	17	
No	44	1	0	
PAD				0.014
Yes	473	33	15	
No	383	19	2	
Cardiac autonomic neuropathy				0.243
Yes	607	37	15	
No	248	12	2	
Gastrointestinal autonomic neuropathy				0.706
Yes	230	13	3	
No	642	39	14	
Bladder autonomic neuropathy				0.192
Yes	431	27	12	
No	460	28	5	
Albuminuria				0.147
Yes	485	38	9	
No	320	14	8	
CKD				0.730
Yes	591	39	11	
No	308	16	6	
Hypertension				0.238
Yes	633	33	11	
No	266	22	6	
Coronary heart diseases				0.528
Yes	215	10	3	
No	684	45	14	
Hyperuricemia				0.263
Yes	116	6	0	
No	783	49	17	
Hyperlipidemia				0.511
Yes	231	17	3	
No	668	38	13	

Values are presented as number. PAD, peripheral arterial disease. CKD, chronic kidney disease.

Nearly half of the foot ulcers belonged to the neuro-ischemic foot ulcers. The first (23.5%) and fifth toes (13.3%) were the main sites of the foot ulcers, followed by heel (12.3%) and dorsum (11.6%) of feet. The foot ulcer size in the minor amputated patients (10.0(3.0-32.4) cm^2^) and the major amputated patients (10.3(3.3-30.0) cm^2^) were significantly larger than that in the non-amputated patients (4.0(1.3-12.0) cm^2^, *P*=0.005). Approximately two thirds of the amputees had foot or toe gangrene. 78(8.0%) of the amputated patients had a history of previous amputations. Osteomyelitis (*P* = 0.006), foot gangrene (*P* < 0.001) and a history of previous amputations (*P* < 0.001) were more common in the patients with amputation than those without amputation. The proportion of patients with Wagner grade 4 and grade 5 foot ulcers in the non-amputation, and minor amputation and major amputation groups were 29.3%, 70.9% and 82.4%, respectively. No DFU patient with Wagner grade 1 and grade 2 was amputated during hospitalization (**Table 3**).

**Table 3:** Comparison of foot-related characteristics among the non-amputation, minor amputation and major amputation groups.

Factor	Non-amputation (N=899)	Minor amputation (N=55)	Major amputation (N=17)	*P* value
Prior ulcer				0.372
Yes	263	21	5	
No	636	34	12	
Prior amputation				<0.001
Yes	61	12	5	
No	838	43	12	
Deformities				0.544
Yes	133	7	1	
No	766	48	16	
Callus				0.087
Yes	310	11	6	
No	589	44	11	
Osteomyelitis				0.006
Yes	422	38	7	
No	429	15	9	
Foot gangrene				<0.001
Yes	263	39	14	
No	636	16	3	
Wagner grade				<0.001
1	72	0	0	
2	170	0	0	
3	393	16	3	
4	250	33	9	
5	14	6	5	

Values are presented as number.

### 3.2 Laboratory tests

The mean HbA1c and FBG levels of the patients with DFU were 8.6 ± 2.3% and 9.0 ± 4.0mmol/L, respectively. The mean Hb, serum albumin and total cholesterol (TC) levels were 113 ± 22.7g/L, 36.2 ± 6.2g/L, and 3.5 ± 1.4 mmol/L, respectively. Compared with the non-amputated patients, the Hb (*P* = 0.004), serum albumin (*P* < 0.001), TC (*P* = 0.016) and UA(*P*=0.001) levels in the amputated patients were statistically lower. Compared with the non-amputated patients, the amputated patients had higher levels of PLT, FIB, WBC counts, NEUT, eGFR and serum CRP, which were the highest in the major-amputated patients. Compared with patients with the non-amputation (0.97 ± 0.28) and minor amputation (0.85 ± 0.33), the patients with major amputation had lower ABI levels (0.76 ± 0.31). In additon, ABI values of 13 cases in the minor amputation group were normal (0.9-1.3). All of the minor amputated patients with normal ABI had osteomyelitis or gangrene, and the sizes of the foot ulcers in more than half of them were larger than 6cm^2^ (**Table 1**).

### 3.3 Risk factors associated with LEAs

Results of the ordinal logistic regression models are shown in **Table 4**. After adjustment of the baseline predictors, a history of prior amputations (*OR*, 5.380; 95% *CI*, 1.847-15.668, *P* = 0.002), foot gangrene (*OR*, 6.854; 95% *CI*, 2.246-20.915, *P* = 0.001) and ABI (*OR*, 0.853; 95% *CI*, 0.733-0.992, *P* = 0.038) significantly associated with LEAs. In addition to eGFR and CRP, a history of prior amputations (*OR*, 10.709; 95% *CI*, 2.871-39.938, *P* = 0.001), foot gangrene (*OR*, 5.625; 95% *CI*, 1.448-7.510, *P* = 0.013) and ABI (*OR*, 0.794; 95% *CI*, 0.649-0.971, *P* = 0.029) significantly associated with LEAs in the Model 2. Finally, in the full Model 3, a history of prior amputations (*OR*, 10.194; 95% *CI*, 2.646-39.279; *P*=0.001), foot gangrene (*OR*, 6.466; 95% *CI*, 1.576-26.539; *P*=0.010) and ABI (*OR*, 0.791; 95% *CI*, 0.639-0.980; *P* = 0.032) were the independent risk factors of LEAs. The ordinal logistic regression model was assessed validity with the test of parallel lines (*P* > 0.05), and significance was confirmed by −2 log likelihood (*P* < 0.001).

**Table 4:** The ordinal logistic regression analysis of major and minor amputation risks in patients with diabetic foot ulcers.

	Model 1 *OR* (95%*CI*)	Model 2 *OR* (95%*CI*)	Model 3 *OR* (95%*CI*)
Prior amputation	5.380(1.847-15.668) ^*^	10.709 (2.871-39.938) ^*^	10.194(2.646-39.279) ^*^
Osteomyelitis	1.254(0.434-3.629)	1.744(0.405-7.510)	1.926(0.443-8.364)
Foot gangrene	6.854(2.246-20.915) ^*^	5.625 (1.448-7.510) ^*^	6.466 (1.576-26.539) ^*^
Ulcer area	1.007(0.996-1.018)	1.011(0.996-1.027)	1.012(0.998-1.027)
Hb	1.010(0.990-1.031)	1.014(0.988-1.041)	1.000(0.994-1.006)
PLT	0.998(0.993-1.003)	0.998(0.993-1.004)	0.998(0.993-1.004)
FIB	/	/	0.642(0.387-1.064)
NEUT	1.015(0.971-1.061)	1.029(0.968-1.094)	1.044(0.975-1.118)
Albumin	0.952(0.873-1.037)	0.950(0.836-1.079)	0.927(0.814-1.002)
TC	0.843(0.595-1.195)	0.778(0.476-1.274)	0.773(0.474-1.260)
UA	0.996(0.991-1.001)	0.993(0.986-1.001)	0.993(0.985-1.002)
eGFR	/	1.025(1.000-1.051)	1.022(0.994-1.011)
CRP	/	0.995(0.984-1.007)	0.999(0.987-1.011)
ABI (per 0.1)	0.853(0.733-0.992) ^*^	0.794(0.649-0.971) ^*^	0.791(0.639-0.980) ^*^

OR, odds ratio; CI, confidence interval; ABI, ankle brachial pressure index. Amputation was defined as an ordinal variable with major amputation, minor amputation and non- amputation. The stepwise ordinal logistic regression was used to identify potential predictors for major and minor amputation and to calculate OR, using the ‘‘non-amputation’’ subgroup as a baseline. Model 1 was adjusted for Hb, PLT, NEUT, albumin, TC, UA, ABI, ulcer area, and the presence of prior amputation, osteomyelitis, foot gangrene. Model 2 was adjusted for eGFR, CRP on the basis of Model 1. *P < 0.05. Model 3 was adjusted for FIB on the basis of Model 2.

### 3.4 Prognosis during hospitalization

The mean hospital stay was 31 (18–56) days, and which of minor (47 (37-63) days) and major amputation groups (57 (38-95) days) were longer than those of non-amputation group (30 (15-55) days, P< 0.001). On discharge, foot ulcers in 240(26.7%) and 94 (9.5%) patients with non-amputation were completely healed and poorly healed, respectively. Foot ulcers of 11(20.0%) and 4(23.5%) patients healed in minor and major amputation group, respectively. 10 (1.0%) patients died during the hospitalization. The main of death causes were myocardial infarction (3 cases), heart failure (3 cases) and respiratory failure (3 cases). One of these died of septic shock after major amputation.

## 4 Discussion

This study showed a comparatively low rate of LEA among the hospitalized patients with DFU in the Diabetic Foot Care Center of a tertiary hospital (7.3%) in China. The previous amputation, foot gangrene and decreased ABI value were independent predictors of LEA. Therefore, it is a great challenge for the practitioners to avoid amputation and re-amputation in the diabetic patients, especially in the elderly and poorly glycemic controlled patients with a previous history of foot ulcer or amputation.

A history of prior foot ulceration was considered as a significant risk factor for amputation (19–21). Furthermore, a prior history of amputation was linked to an increased risk of major adverse limb events (22). One meta-analysis about risk of major amputation in the DFU patients showed that hypertension, ischemic heart disease, cerebrovascular disease and peripheral vascular disease were identified as the predisposing factors for major amputation (10).The FIELD study indicated that previous cardiovascular disease, microvascular disease, previous non-traumatic amputation or skin ulcer, smoking, and longer duration of diabetes were more frequent in the amputated patients than in the non-amputated patients (23). Therefore, the diabetic patients experienced non-traumatic lower-limb amputations were multifactorial.

It appears that PAD was more common in the minor (63.5%) and major (88.2%) amputated patients than the non-amputated patients (55.3%) (*P*=0.014) in this study. A study consisting of 3892 type 2 diabetes patients with a first-time diagnosis of diabetic foot syndrome in German showed that the presence of PAD was the strongest independent predictor of LEA in the DFU patients (*HR*, 5.13; *CI*: 4.27–6.16) (24). Another prospective single-center study in German showed that perfusion status of foot, and ulcer extent and depth were the risk factors of LEA according to the PEDIS classification (25). Lower extremity artery stenosis or occlusion was considered as a risk factor for amputation in the DFU patients (26, 27). ABI was a simple and non-invasive method to screen PAD. In this study, the mean values of ABI in the major and minor amputation groups were 0.76 and 0.85, respectively. The decreased ABI value was a strong predictor for LEA. Another prospective single-center study in China also suggested that low ABI were significantly associated with an increased risk of LEA (28). The SEASON study in Japan suggested that ABI <0.4 was the strongest risk factor for amputation of the diabetic patients with PAD (29). In the FIELD study, ABI >0.52 increased a rate of limb preservation in the patients with chronic limb-threatening ischemia (23). Thus, IWGDF recommended that a screening ABI should be performed in the diabetic patients who had symptoms or signs of PAD or who were over than older than 50 years old (30). Actually, ABI was not completed reliable on diagnosis of PAD in the diabetic patients. ABI could falsely elevate due to calcification of arterial media (31). Falsely high ABI was an independent predictor of major amputation in the patients with chronic limb ischemia (32). In addition, our study showed that LEA occurred even in the DFU patients with normal ABI values, especially in the minor amputated patients. A Korean study found that 28.7% of patients had normal ABI ranging from 0.91 to 1.40 but were diagnosed with PAD using color doppler ultrasonography (33). Our previous study showed that 19.8% of limbs in the patients with diabetic foot disease had normal ABI values (0.91-1.3). However, digital subtraction arteriography showed that 72.2% of the lower limbs with normal ABI had occlusion of at least one artery below knees (34). This could be explained by extensive distribution and multiple segments of atherosclerotic lesions in below-the-knee arteries or formation of collaterals. Therefore, ABI could underestimate PAD in the DFU patients and color doppler ultrasound was usually necessary for further diagnosis of PAD in the diabetic patients with foot ulcers.

We found that the hospitalized DFU patients with foot gangrene had an approximately 6.5-fold higher risk of amputation. Foot gangrene was caused by deficient blood supply to tissues due to arterial stenosis or occlusion that further led to localized necrosis and tissue death. Mortality rate was significantly high after major amputation. A study in Tanzania revealed that the overall mortality rates for amputees and non-amputees were similar (29%), but patients with severe foot ulcers (Wagner grade ≥ 4) who did not undergo surgery had the highest mortality rate (54%) during hospitalization (35). Another retrospective study in Finland showed that after a major amputation, the one- and five-year overall survival rates of the diabetic patients with foot infection were 41.7% and 8.3%, respectively (36). Rapid revascularization, either endovascular or open vascular surgery, could reduce the risk of amputation in DFU patients with the PAD (37). The incidence of gangrene decreased from 14.7% to 11.3% (*P*<0.001) with a concomitant increase in vascular interventions (6.2% to 19.5%, *P*<0.001). Therefore, it is critical to take effective measures to improve blood supply of the gangrene foot early as much as possible in order to effectively reduce the amputation plane of the patients with severe foot ulcers, even avoid major amputation.

Prothrombotic state was more pronounced in the amputated patients than those in the non-amputated patients, which implied increased coagulation, impaired fibrinolysis, and endothelial dysfunction (38). This was illustrated by higher fibrinogen levels in the amputated patients compared with the non-amputated patients from this study and other studies (39, 40). Wang et al. suggested that fibrinogen was an independent risk factor of LEA in the DFU patients (39). Plasma fibrinogen level >300.4 mg% (100% sensitivity, 99.2% specificity) was correlated with a high risk of amputation in DFU (41). Another study showed a fibrinogen cut-off value of 5.13g/L indicated the possible amputation with a sensitivity of 81.8% and a specificity of 78.9% (positive predictive value 78.6%, negative predictive value 89.0%) (40). Therefore, early anticoagulant treatment undoubtedly improve prognosis of DFU.

Foot ulcer infection was closely associated with the increased amputation rate. In routine clinical practice, WBC, PLT, and CRP levels were used to determine procession of DFU (42). A prospective study in Turkey showed that 33.2% of 126 cases with diabetic foot infection (DFI) underwent amputation (43). Approximately 50% of DFU patients could develop DFI, which was diagnosed on the basis of clinical characteristics (44). Inflammatory biomarkers such as WBC, Neutrophils, CRP, IL-6, PCT and ESR could be used to distinguish between non-infection and mild infection, indicate severity of foot ulcer infection and monitor response of anti-infective therapy. Therefore, the inflammatory markers were reported to be a strong predictor of amputation (45, 46). In our study, compared with the non-amputated patients, the DFU patients with minor and major amputations had higher levels of WBC counts, NEUT, and serum CRP, which were higher in the major amputees than the minor amputees. Foot gangrene and osteomyelitis affected roughly one-third and one-half of the amputees, respectively. One meta-analysis showed that osteomyelitis (OR: 4.5), neuro-ischemic DFI (OR: 3.06), severe infection (OR: 3.12), leukocytosis (OR: 1.76), mean ESR (SMD: 0.5), mean CRP (SMD: 0.8), tissue culture positivity (OR: 1.61), and isolation of Gram-negative bacteria from tissue culture (OR: 1.5) were predictors of amputation in DFI (19). PCT was a diagnostic marker of bacterial infection. Another meta-analysis revealed that PCT>0.5 ng/ml was an independent predictor of major amputation (OR 3.3) and mortality (OR 4.13) in the DFI patients with CLI (47). WBC, ESR and CRP were non-specific inflammatory biomarkers. Therefore, testing for the inflammatory biomarkers in the DFU patients could help early identify diagnosis of DFI and monitor therapeutic response after anti-infective treatment.

The process of wound healing required adequate nutrient supply to the tissue, which could be hampered by circulatory compromise and rapid protein loss (48, 49). Malnutrition is highly prevalent among the DFU patients (50). Serum albumin and Hb were used to evaluate the nutritional status of human body. Compared with the non-amputated patients, the amputated patients had significantly lower Hb, serum albumin and TC levels. A study enrolling 3654 patients with DFU revealed that Hb and plasma albumin were the independent factors of major amputation (21). There was no definitive evidence to confirm the close relationship between malnutrition and amputation in the DFU patients, but protein-energy wasting was common in the DFU patients with severe infection. Thus, the clinicians should focus on the nutritional status of the DFU patients and correct their anemia and hypoalbuminemia as soon as possible in order to improve general conditions of the patients and promote wound healing.

A multicenter study revealed that the diabetic patient with even moderate CKD(eGFR<60ml/min per 1.73m^2^) had an increased risk for DFU and LEA (51). The eGFR<30ml/min per 1.73m^2^ in DFU patients with osteomyelitis was an independent predictor for amputation and healing failure (52). However, in this study, we found the mean eGFR value in the amputated patients was over 60 mL/min per 1.73 m^2^, which was higher than that in the non-amputees. Although we could not fully explain why the amputated patients had higher eGFR compared with the non-amputated patients, glomerular hyperfiltration due to hyperglycemia and adequate rehydration for the amputated patients was the possible reason.

Most of patients in our study had eventually good therapeutic effects with low amputation and mortality rates, highlighting the importance of the multidisciplinary intervention. However, the study had several limitations. It was a retrospective study from single medical center, which could lead to selection bias. Clinical data of some patients were incomplete. The number of LEA outcomes was low which was good for the patients, but reduced our sample size. In addition, treatment strategies, e.g., revascularization (surgical or endovascular), statin therapy, was not considered, which may render some of risk estimates unstable.

## 5 Conclusion

The DFU inpatients with LEA were older with long duration of diabetes, poorly glycemic control, malnutrition, high prevalence of PAD, severe foot ulcers and infection, and longer hospital stays. A history of prior amputation, foot gangrene and a low ABI level were the independent predictors of LEA. However, normal ABI could not exclude PAD and LEA was caused by multiple factors which should be concerned. Therefore, multidisciplinary diagnosis and treatment of DFU is essential to avoid amputation of the DFU patients.

## Data availability statement

The original contributions presented in the study are included in the article. Further inquiries can be directed to the corresponding author.

## Ethics statement

The studies involving human participants were reviewed and approved by The Ethics Committee of the West China Hospital, Sichuan University. The patients/participants provided their written informed consent to participate in this study.

## Author contributions

HG ontributes in the proposal preparation, analysis and writes up of the manuscript. HG and CW revised the manuscript. CW contributed to study design and analysis. DC, YG, and XR contributed to the design of the research protocol. HG, YR, ZL, PZ, YL, RB, and LC contributed to the collection of the clinical data. All authors contributed to and approved the final manuscript for publication.

## References

[1] Schaper NC Apelqvist J Bakker K. The international consensus and practical guidelines on the management and prevention of the diabetic foot. Curr Diabetes Rep (2003) 3(6):475–9. doi: 10.1007/s11892-003-0010-4 14611743

[2] Zhang P Lu J Jing Y Tang S Zhu D Bi Y. Global epidemiology of diabetic foot ulceration: A systematic review and meta-analysis (†). Ann Med (2017) 49(2):106–16. doi: 10.1080/07853890.2016.1231932 27585063

[3] Franz D Zheng Y Leeper NJ Chandra V Montez-Rath M Chang TI. Trends in rates of lower extremity amputation among patients with end-stage renal disease who receive dialysis. JAMA Internal Med (2018) 178(8):1025–32. doi: 10.1001/jamainternmed.2018.2436 PMC6143114 29987332

[4] Jiang Y Ran X Jia L Yang C Wang P Ma J. Epidemiology of type 2 diabetic foot problems and predictive factors for amputation in China. Int J lower extremity wounds. (2015) 14(1):19–27. doi: 10.1177/1534734614564867 25573978

[5] Skrepnek GH Armstrong DG Mills JL. Open bypass and endovascular procedures among diabetic foot ulcer cases in the United States from 2001 to 2010. J Vasc surgery. (2014) 60(5):1255–65. doi: 10.1016/j.jvs.2014.04.071 25017514

[6] Rigato M Pizzol D Tiago A Putoto G Avogaro A Fadini GP. Characteristics, prevalence, and outcomes of diabetic foot ulcers in Africa. A systemic Rev meta-analysis. Diabetes Res Clin practice. (2018) 142:63–73. doi: 10.1016/j.diabres.2018.05.016 29807105

[7] Ha Van G Amouyal C Bourron O Aubert C Carlier A Mosbah H. Diabetic foot ulcer management in a multidisciplinary foot centre: One-year healing, amputation and mortality rate. J Wound Care (2020) 29(8):464–71. doi: 10.12968/jowc.2020.29.8.464 32804035

[8] Wukich DK Raspovic KM Suder NC. Patients with diabetic foot disease fear major lower-extremity amputation more than death. Foot Ankle Spec (2018) 11(1):17–21. doi: 10.1177/1938640017694722 28817962

[9] Costa RHR Cardoso NA Procópio RJ Navarro TP Dardik A de Loiola Cisneros L. Diabetic foot ulcer carries high amputation and mortality rates, particularly in the presence of advanced age, peripheral artery disease and anemia. Diabetes Metab syndrome. (2017) 11 Suppl 2:S583–s7. doi: 10.1016/j.dsx.2017.04.008 28465149

[10] Shin JY Roh SG Sharaf B Lee NH. Risk of major limb amputation in diabetic foot ulcer and accompanying disease: A meta-analysis. J plastic reconstructive aesthetic surgery: JPRAS. (2017) 70(12):1681–8. doi: 10.1016/j.bjps.2017.07.015 28865989

[11] Hinchliffe RJ Forsythe RO Apelqvist J Boyko EJ Fitridge R Hong JP. Guidelines on diagnosis, prognosis, and management of peripheral artery disease in patients with foot ulcers and diabetes (IWGDF 2019 update). Diabetes/metabolism Res Rev (2020) 36 Suppl 1:e3276. doi: 10.1002/dmrr.3276 31958217

[12] Bakker K Apelqvist J Lipsky BA Van Netten JJ Schaper NC. The 2015 IWGDF guidance on the prevention and management of foot problems in diabetes. Int Wound J (2016) 13(5):1072. doi: 10.1111/iwj.12496 26345364 PMC7949980

[13] Pop-Busui R Boulton AJM Feldman EL Bril V Freeman R Malik RA. Diabetic neuropathy: A position statement by the American diabetes association. Diabetes Care (2017) 40(1):136–54. doi: 10.2337/dc16-2042 PMC6977405 27999003

[14] Tesfaye S Boulton AJ Dyck PJ Freeman R Horowitz M Kempler P. Diabetic neuropathies: Update on definitions, diagnostic criteria, estimation of severity, and treatments. Diabetes Care (2010) 33(10):2285–93. doi: 10.2337/dc10-1303 PMC2945176 20876709

[15] Boulton AJM Vinik AI Arezzo JC Bril V Feldman EL Freeman R. Diabetic neuropathies: A statement by the American diabetes association. Diabetes Care (2005) 28(4):956–62. doi: 10.2337/diacare.28.4.956 15793206

[16] Gatopoulou A Papanas N Maltezos E. Diabetic gastrointestinal autonomic neuropathy: Current status and new achievements for everyday clinical practice. Eur J Internal Med (2012) 23(6):499–505. doi: 10.1016/j.ejim.2012.03.001 22863425

[17] Stevens PE Levin A. Evaluation and management of chronic kidney disease: synopsis of the kidney disease: improving global outcomes 2012 clinical practice guideline. Ann Internal Med (2013) 158(11):825–30. doi: 10.7326/0003-4819-158-11-201306040-00007 23732715

[18] Bury DC Rogers TS Dickman MM. Osteomyelitis: Diagnosis and treatment. Am Family physician. (2021) 104(4):395–402. 34652112

[19] Sen P Demirdal T Emir B. Meta-analysis of risk factors for amputation in diabetic foot infections. Diabetes/metabolism Res Rev (2019) 35(7):e3165. doi: 10.1002/dmrr.3165 30953392

[20] Lin C Liu J Sun H. Risk factors for lower extremity amputation in patients with diabetic foot ulcers: A meta-analysis. PloS One (2020) 15(9):e0239236. doi: 10.1371/journal.pone.0239236 32936828 PMC7494323

[21] Lu Q Wang J Wei X Wang G Xu Y. Risk factors for major amputation in diabetic foot ulcer patients. Diabetes Metab syndrome obesity: Targets Ther (2021) 14:2019–27. doi: 10.2147/DMSO.S307815 PMC8106455 33976562

[22] Weissler EH Clare RM Lokhnygina Y Buse JB Goodman SG Katona B. Predicting major adverse limb events in individuals with type 2 diabetes: Insights from the EXSCEL trial. Diabetic medicine: J Br Diabetic Assoc (2021) 38(10):e14552. doi: 10.1111/dme.14552 PMC8429063 33690915

[23] Rajamani K Colman PG Li LP Best JD Voysey M D’Emden MC. Effect of fenofibrate on amputation events in people with type 2 diabetes mellitus (FIELD study): A prespecified analysis of a randomised controlled trial. Lancet (London England). (2009) 373(9677):1780–8. doi: 10.1016/S0140-6736(09)60698-X PMC2687887 19465233

[24] Pscherer S Dippel FW Lauterbach S Kostev K. Amputation rate and risk factors in type 2 patients with diabetic foot syndrome under real-life conditions in Germany. Primary Care diabetes. (2012) 6(3):241–6. doi: 10.1016/j.pcd.2012.02.004 22445058

[25] Hüsers J Hafer G Heggemann J Wiemeyer S John SM Hübner U. Predicting the amputation risk for patients with diabetic foot ulceration - a Bayesian decision support tool. BMC Med Inf decision making. (2020) 20(1):200. doi: 10.1186/s12911-020-01195-x PMC7446175 32838777

[26] Morbach S Furchert H Gröblinghoff U Hoffmeier H Kersten K Klauke GT. Long-term prognosis of diabetic foot patients and their limbs: Amputation and death over the course of a decade. Diabetes Care (2012) 35(10):2021–7. doi: 10.2337/dc12-0200 PMC3447849 22815299

[27] Winkley K Stahl D Chalder T Edmonds ME Ismail K. Risk factors associated with adverse outcomes in a population-based prospective cohort study of people with their first diabetic foot ulcer. J Diabetes its complications. (2007) 21(6):341–9. doi: 10.1016/j.jdiacomp.2007.09.004 17967704

[28] Sun JH Tsai JS Huang CH Lin CH Yang HM Chan YS. Risk factors for lower extremity amputation in diabetic foot disease categorized by Wagner classification. Diabetes Res Clin practice. (2012) 95(3):358–63. doi: 10.1016/j.diabres.2011.10.034 22115502

[29] Higashi Y Miyata T Shigematsu H Origasa H Fujita M Matsuo H. Evaluation of risk factors for major amputation in patients with diabetes and peripheral artery disease receiving antiplatelet Therapy - *Post hoc* analysis of a prospective observational multicenter cohort study (SEASON). Circ journal: Off J Japanese Circ Society. (2019) 83(9):1929–36. doi: 10.1253/circj.CJ-19-0088 31292312

[30] Schaper NC Van Netten JJ Apelqvist J Lipsky BA Bakker K. Prevention and management of foot problems in diabetes: A summary guidance for daily practice 2015, based on the IWGDF guidance documents. Diabetes/metabolism Res Rev (2016) 32 Suppl 1:7–15. doi: 10.1002/dmrr.2695 26335366

[31] Langham MC Floyd TF Mohler ER 3rd Magland JF Wehrli FW. Evaluation of cuff-induced ischemia in the lower extremity by magnetic resonance oximetry. J Am Coll Cardiol (2010) 55(6):598–606. doi: 10.1016/j.jacc.2009.08.068 20152564 PMC2833093

[32] Silvestro A Diehm N Savolainen H Do DD Vögelea J Mahler F. Falsely high ankle-brachial index predicts major amputation in critical limb ischemia. Vasc Med (London England). (2006) 11(2):69–74. doi: 10.1191/1358863x06vm678oa 16886836

[33] Hur KY Jun JE Choi YJ Lee YH Kim DJ Park SW. Color Doppler ultrasonography is a useful tool for diagnosis of peripheral artery disease in type 2 diabetes mellitus patients with ankle-brachial index 0. 91 to 1.40. Diabetes Metab J (2018) 42(1):63–73. doi: 10.4093/dmj.2018.42.1.63 29504306 PMC5842302

[34] Chen DW Lu WS Wang C Jiao H Song YX Chen LH. [Digital subtract arteriographic (DSA) characteristics of lower extremities and ankle-brachial index in patients with diabetic feet]. J Sichuan Univ Med Sci edition. (2010) 41(4):731–3, 50. 20848802

[35] Gulam-Abbas Z Lutale JK Morbach S Archibald LK. Clinical outcome of diabetes patients hospitalized with foot ulcers, dar es salaam, Tanzania. Diabetic medicine: J Br Diabetic Assoc (2002) 19(7):575–9. doi: 10.1046/j.1464-5491.2002.00740.x 12099961

[36] Vuorlaakso M Kiiski J Salonen T Karppelin M Helminen M Kaartinen I. Major amputation profoundly increases mortality in patients with diabetic foot infection. Front surgery. (2021) 8:655902. doi: 10.3389/fsurg.2021.655902 PMC8120024 33996886

[37] Butt T Lilja E Elgzyri T Apelqvist J Gottsäter A Engström G. Amputation-free survival in patients with diabetic foot ulcer and peripheral arterial disease: Endovascular versus open surgery in a propensity score adjusted analysis. J Diabetes its complications. (2020) 34(5):107551. doi: 10.1016/j.jdiacomp.2020.107551 32061519

[38] Walinjkar RS Khadse S Kumar S Bawankule S Acharya S. Platelet indices as a predictor of microvascular complications in type 2 diabetes. Indian J Endocrinol Metab (2019) 23(2):206–10. doi: 10.4103/ijem.IJEM_13_19 PMC6540898 31161104

[39] Wang L Li Q Chen X Wang Z. Clinical characteristics and risk factors of lower extremity amputation in patients with diabetic foot. Pakistan J Med Sci (2022) 38(8):2253–8. doi: 10.12669/pjms.38.8.5635 PMC9676613 36415262

[40] Li XH Guan LY Lin HY Wang SH Cao YQ Jiang XY. Fibrinogen: A marker in predicting diabetic foot ulcer severity. J Diabetes Res (2016) 2016:2358321. doi: 10.1155/2016/2358321 28044140 PMC5156809

[41] Rattan R Nayak D. High levels of plasma malondialdehyde, protein carbonyl, and fibrinogen have prognostic potential to predict poor outcomes in patients with diabetic foot wounds: A preliminary communication. Int J lower extremity wounds. (2008) 7(4):198–203. doi: 10.1177/1534734608324124 18815200

[42] Wong KL Nather A Liang S Chang Z Wong TT Lim CT. Clinical outcomes of below knee amputations in diabetic foot patients. Ann Acad Medicine Singapore. (2013) 42(8):388–94. doi: 10.47102/annals-acadmedsg.V42N8p388 24045374

[43] Uysal S Arda B Taşbakan MI Çetinkalp Ş Şimşir IY Öztürk AM. Risk factors for amputation in patients with diabetic foot infection: a prospective study. Int Wound J (2017) 14(6):1219–24. doi: 10.1111/iwj.12788 PMC7950123 28722354

[44] Mponponsuo K Sibbald RG Somayaji R. A comprehensive review of the pathogenesis, diagnosis, and management of diabetic foot infections. Adv skin Wound Care (2021) 34(11):574–81. doi: 10.1097/01.ASW.0000791876.10485.d4 34669660

[45] Lin CW Hsu LA Chen CC Yeh JT Sun JH Lin CH. C-reactive protein as an outcome predictor for percutaneous transluminal angioplasty in diabetic patients with peripheral arterial disease and infected foot ulcers. Diabetes Res Clin practice. (2010) 90(2):167–72. doi: 10.1016/j.diabres.2010.08.002 20822820

[46] Li X Xiao T Wang Y Gu H Liu Z Jiang Y. Incidence, risk factors for amputation among patients with diabetic foot ulcer in a Chinese tertiary hospital. Diabetes Res Clin practice. (2011) 93(1):26–30. doi: 10.1016/j.diabres.2011.03.014 21466901

[47] Meloni M Izzo V Giurato L Brocco E Ferrannini M Gandini R. Procalcitonin is a prognostic marker of hospital outcomes in patients with critical limb ischemia and diabetic foot infection. J Diabetes Res (2019) 2019:4312737. doi: 10.1155/2019/4312737 31485450 PMC6710766

[48] Halloran CM Slavin JP. Pathophysiology of wound healing. Surg (Oxford) (2002) 20(5):i–v. doi: 10.1383/surg.20.5.0.14629

[49] Litchford MD. Chapter 8 - nutritional issues in the patient with diabetes and foot ulcers. In: Bowker JH Pfeifer MA, editors. Levin And O’Neal’s the diabetic foot, Seventh Edition. Philadelphia, PA: Mosby Elsevier (2008). p. 199–217.

[50] Lauwers P Dirinck E Van Bouwel S Verrijken A Van Dessel K Van Gils C. Malnutrition and its relation with diabetic foot ulcer severity and outcome: A review. Acta clinica Belgica. (2022) 77(1):79–85. doi: 10.1080/17843286.2020.1800315 32727304

[51] Margolis DJ Hofstad O Feldman HI. Association between renal failure and foot ulcer or lower-extremity amputation in patients with diabetes. Diabetes Care (2008) 31(7):1331–6. doi: 10.2337/dc07-2244 PMC2453658 18390800

[52] Zhang J Chen D Li X Ding M Xu J Wang M. The association between estimated glomerular filtration rate and prognosis in patients with diabetic foot osteomyelitis. Int Wound J (2022) 19(7):1650–7. doi: 10.1111/iwj.13765 PMC9615297 35080116

